# Does Involving Parents in Soil Sampling Identify Causes of Child Exposure to Lead? A Case Study of Community Engagement in Mining‐Impacted Towns in Peru

**DOI:** 10.1029/2019GH000200

**Published:** 2019-08-23

**Authors:** Franziska C. Landes, Jennifer Inauen, Johny Ponce‐Canchihuamán, Kathie Markowski, Tyler K. Ellis, Alexander van Geen

**Affiliations:** ^1^ Lamont‐Doherty Earth Observatory Columbia University Palisades NY USA; ^2^ Department of Earth and Environmental Sciences Columbia University New York NY USA; ^3^ Institute of Psychology University of Bern Bern Switzerland; ^4^ Center for Research in Environmental Health Lima Peru; ^5^ Facultad de Salud Publica y Administracion Universidad Peruana Cayetano Heredia Lima Peru

**Keywords:** soil, lead (Pb), Peru, child blood‐lead levels (BLL), mining towns, community testing

## Abstract

Over a million people in Peru may be exposed to lead (Pb) due to past or present mining‐related activities; however, neither soil Pb nor blood Pb are routinely monitored throughout the country. Because little is known about Pb contamination in smaller mining‐impacted towns, soil Pb was mapped in four such towns with a portable X‐ray fluorescence analyzer in 2015. The roadside mapping delineated hotspots of highly contaminated soil (1,000–6,000 mg/kg Pb) in two of the towns. The local health department, provided with a LeadCare II analyzer, then measured blood‐Pb levels >5 in 65% and >10 μg/dL in 15% of children (*n* = 200) up to 6 years of age in these same four communities. There were no clear relations between child blood‐Pb levels and Pb levels in soil samples collected inside (*n* = 50) or outside the home (*n* = 50). Increased child blood Pb was associated with decreased level of cleanliness of parent clothing (*n* = 136) and shoes (*n* = 138), linking a possible behavioral factor for transferring contaminated soil and dust to children. In order to explore individual exposure and variations in soil Pb, 10 parents of children with blood Pb >10 μg/dL and 10 parents of children with blood Pb <5 μg/dL were invited to collect soil samples in areas where their children play and screen it for Pb using a color‐based field procedure. Importantly, parents identified a new hotspot of Pb contamination that had been missed by the previous portable X‐ray fluorescence soil mapping. The findings highlight the feasibility and value of involving families impacted by environmental contamination to identify and reduce environmental health risk.

## Key Points

- Parents in four small mining‐impacted towns in Peru successfully collected soil samples from areas where their children play
- Parents analyzed samples with trained help using a field procedure that led to the identification of a new hotspot of Pb contamination
- Child blood‐Pb was associated with parent shoe and clothing cleanliness as well as education level, but not with Pb in soil

## 1 Introduction

The toxicity of lead (Pb) is widely recognized; lead has been linked to childhood neurological impairment (Lanphear et al., 2005; Needleman & Gatsonis, 1990; Tong et al., 2000; World Health Organization, 2010) and cardiovascular disease later in life (Lanphear et al., 2018; Navas‐Acien et al., 2007). Children under the age of 6 are at highest risk of Pb exposure as they explore their environment by crawling on the ground and placing dusty toys and hands into their mouths. Children show reduced test scores and greater risk of learning disabilities even at blood lead levels (BLLs) previously considered safe (Lanphear et al., 2005). To reflect this, the U.S. Centers for Disease Control and Prevention (CDC) in 2012 replaced its BLL standard of 10 μg/dL with a reference level of 5 μg/dL. This reference level is based on the 97.5th percentile of children tested in the 2010 National Health and Nutritional Examination Study. Because exposure in the United States continues to decline, this reference level would have to be updated to 3.5 μg/dL based on the 2015 National Health and Nutritional Examination Study (Tsoi et al., 2016; U.S. CDC, 2012, 2017).

Child BLLs have been associated with soil‐Pb levels (Lanphear et al., 1998; Mielke & Reagan, 1998) and resuspended Pb in air (Laidlaw & Filippelli, 2008; Zahran et al., 2013). A pooled analysis by Lanphear et al. (1998) of 12 studies found Pb loading in house dust, collected by wipe method (mass per area) was the major contributor to BLL in children age 6 to 36 months. Soil‐Pb concentrations also predicted child BLL levels in this study as did age, mouthing behaviors, and race. Indoor dust‐Pb loading also contributed more to child BLL than soil Pb. Child BLLs, which peak in summer months, have been linked to soil Pb through atmospheric conditions and the resuspension of soil‐Pb into the air, with children age 2 and younger the most impacted (Zahran et al., 2013). The review by Mielke and Reagan (1998) of studies with BLL and possible contributors of air, dust, soil, water, food, and paint demonstrated that 42 of the 46 studies with soil‐Pb information found a positive correlation between BLL and soil Pb (Mielke & Reagan, 1998). An analysis in Bunker Hill, an old smelting and mining area in Idaho, found that home‐soil and neighborhood‐soil Pb levels were most related to BLLs of younger children, while community‐wide soils had the largest impact on BLL overall and were highest for children 5 to 6 years old (von Lindern et al., 2003).

While BLLs in the United States have dropped precipitously from 99.8% >5 μg/dL (1976–1980; Pirkle et al., 1994) to 0.5% (2013–2014; Tsoi et al., 2016), Pb poisoning is still a major health issue throughout many low‐ and middle‐income countries (Landrigan et al., 2018; Tong et al., 2000). In Peru, for instance, the Ministry of Health tested 233 children in the mining city of Cerro de Pasco in 2005 and found BLLs in 86% of children >10 μg/dL and 22% >20 μg/dL (Astete et al., 2005). Elevated BLLs are likely found throughout Peru: A national assessment calculated that 1.6 million people live within 5 km of an active mine, former mine, ore processor, or smelter (van Geen et al., 2012). Testing conducted in 2014 in the smelter town of La Oroya found BLLs in 58% of 335 children >10 μg/dL (Red Salud, 2014). Other studies in Peru have found BLLs associated with traffic levels in police officers (Mormontoy et al., 2006) and mineral ore storage in Callao (Hernandez Avila, 1999; Naeher et al., 2003; Vega‐Dienstmaier et al., 2006).

Although the more infamous cases of Pb contamination in Peru have been described in cities like Cerro de Pasco (Conklin et al., 2008), La Oroya (Castro‐Bedriñana et al., 2013) and Callao (Naeher et al., 2003), little is known about smaller towns that are also impacted by Pb‐contaminating industries such as smelting, mining, or battery recycling. Many such towns neighbor the larger industrial centers or are situated along the major transportation routes for ore concentrates. In this study, we investigated the extent of soil‐Pb contamination in four of these smaller potentially impacted towns in the Huarochiri Province of central Peru with a roadside mapping by X‐ray fluorescence. The results showing two areas highly contaminated with Pb were presented to community members and municipalities. A group of local parents was then trained to collect soil samples in areas where their children play, and they participated in the screening of their soil samples for Pb using a simple test. We show that local participation proved to be valuable because sampling by parents revealed a previously unidentified contaminated area. We also explore possible associations of soil, dust, and survey results obtained as part of the study with child BLLs obtained by the local health department.

## 2 Materials and Methods

All protocols involving human subjects research were approved by the CU Institutional Review Board and the Peruvian Institutional Review Board, PRISMA. The project was also presented to and endorsed by the local municipalities and health centers.

Fieldwork was conducted with the local not‐for‐profit organization Center for Research in Environmental Health (CREEH) based in Lima, Peru. We regularly met with local health departments and municipalities before, during, and after the soil sampling.

### 2.1 Instrumental Analysis

Surface soil samples were analyzed in situ with a portable, X‐ray fluorescence (XRF) instrument (InnovX Delta Premium) and ex situ with a benchtop stand for the instrument. For ex situ analysis, sieved samples (<1 mm) in 20‐ml scintillation vials were sealed with plastic wrap and inverted on the XRF. Analysis time in the benchtop stand was 20 s at each of three incident‐beam energies in the instrument's standard soil mode. When measuring soil in situ in the field, the analysis time was reduced to 10 s per incident‐beam. Filtered soil extracts as well as home soil and/or dust samples were also analyzed by the XRF in the benchtop stand.

X‐ray fluorescence is increasingly used to screen soils, as in EPA Test Method 6200 (U.S. EPA, 2015), because of its low cost per sample when compared to acid digestions and mass spectrometer analysis, and the soil‐Pb results correlate highly with digest results (Markey et al., 2008; Radu & Diamond, 2009). In this work, the XRF's internal calibration and data reduction was confirmed by repeat analysis of Standard Reference Material soils from the U.S. National Institute of Standards and Technology (NIST) with high (5,532 mg/kg Pb in NIST 2710), medium (1,162 mg/kg Pb in NIST 2711), and low (18.9 mg/kg Pb in NIST 2709) concentrations of Pb. The XRF results were within 10% of NIST published values for standards 2711 (*n* = 55) and 2710 (*n* = 8). The XRF overestimated, on average, the 18.9 mg/kg from NIST 2709 (*n* = 7) as 22.9 (120%) mg/kg.

Recent work has shown that XRF analysis of Pb in liquid extracts highly correlates with analysis by inductively coupled plasma mass spectrometer (ICP‐MS; Landes et al., 2019), which was also seen for samples analyzed in the present study (Figure S1 in the supporting information). Concentrations of Pb in water samples were analyzed in the laboratory by high‐resolution ICP‐MS (Thermo Scientific Element II); all field‐procedure extracts were also analyzed by ICP‐MS for confirmation. The ICP‐MS procedure was verified by analyzing NIST reference materials 1640A (*n* = 15), which was within 6% of its published value of 12.101 μg/L Pb, and 1643F (*n* = 15), which was within 12% of its published value of 19.63 μg/L Pb. All ICP‐MS dilution and vial blanks contained <1 μg/L Pb.

### 2.2 Site Description

**Figure 1:**
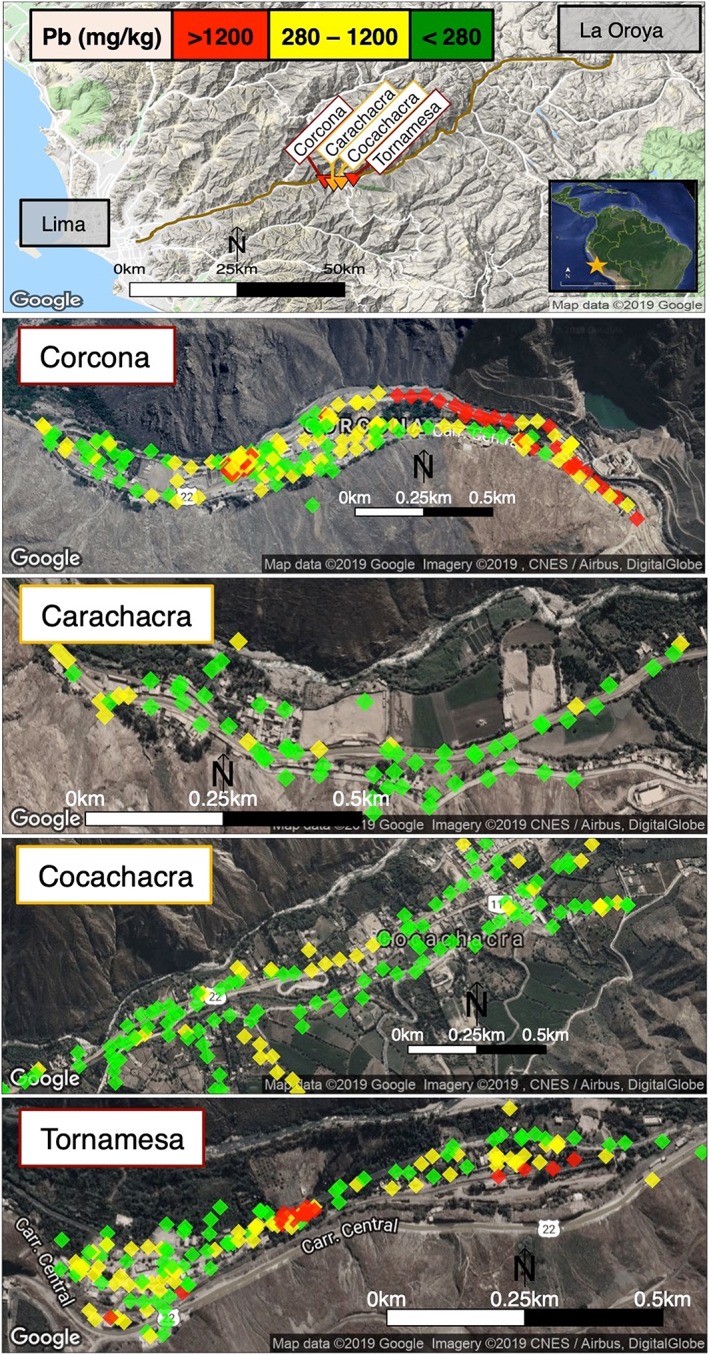
Study location in Peru, 40‐ to 60‐km east of Lima. Soil sample locations of roadside XRF mapping are shown in the four towns and total‐Pb concentration as measured by XRF are indicated in green (<280 mg/kg Pb), yellow (280–1,200 mg/kg Pb), and red (>1,200 mg/kg Pb). The threshold of 280 mg/kg Pb was chosen by doubling the Peruvian soil standard of 140 mg/kg.

The study took place in the four neighboring communities of Corcona, Carachacra, Cocachacra, and Tornamesa approximately 40‐ to 60‐km east of Lima (−11.914°W, −76.546°S) at an elevation of 1,400 ± 150 m. The sequence of four towns follows a narrow valley along the railroad and the Carretera Central, the main highway connecting the smelter town of La Oroya to the port of Callao, north of the capital of Lima (Figure 1). These four towns were chosen due to their location along this major thoroughfare, based on the assumption that railcars and trucks carrying mineral ore concentrate provided a possible source of Pb contamination. In addition, on the eastern end of Corcona, approximately 50‐km east of Lima, volcanic‐hosted massive‐sulfide deposits were mined for barite (BaSO_4_), galena (PbS), pyrite (FeS_2_), sphalerite ((Zn,Fe)S), and chalcopyrite (CuFeS_2_). Mining of barite deposits peaked in the late 1970s, and mining of Zn and Pb deposits increased in the early to mid‐1980s (Vidal, 1987). The study area receives only 250 mm of rainfall annually, leading to dry conditions favorable for soil resuspension.

### 2.3 Local Interviews/Surveys

**Figure 2:**
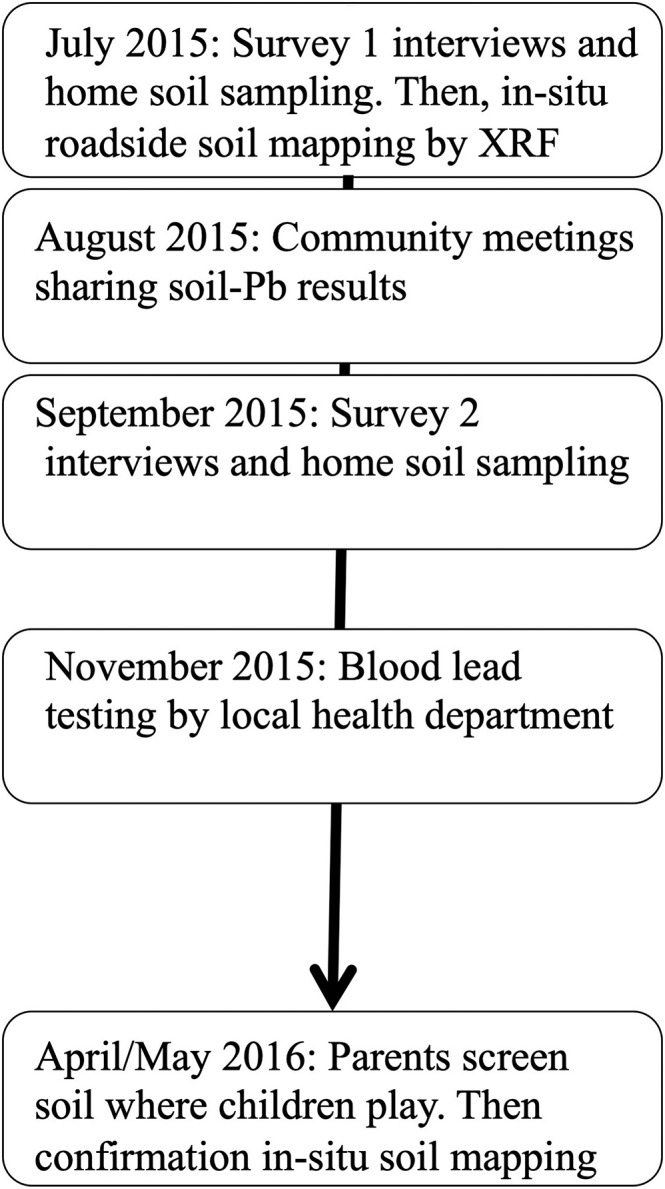
Study timeline from 2015 to 2016 including interview surveys, home soil sampling, roadside soil mapping by XRF, and parent soil screening.

In July 2015, CREEH staff trained five interviewers to survey adult primary caregivers, hereafter referred to as parents, of children 6 years old and younger (Figure 2 and Table 1). After posting flyers throughout the four towns, potential participants were contacted door to door. Interviewers surveyed 149 parents in July (Survey 1) and revisited 137 eight weeks later (Survey 2), approximately 4 weeks after a community meeting (see section 2.7). As far as we can tell, we contacted all parents of children under 6 years of age who were at home during Survey 1. We were unable to find a complete list of parents; the best records were from the governmental Vaso‐de‐Leche (glass of milk) program, which did not include higher income families. The survey included questions about the health impacts of Pb exposure, possible locations of Pb contamination, demographic information, questions assessing exposure‐relevant behaviors, and factors that could impact those behaviors (Mosler, 2012). Interviewers also recorded observations such as children playing on the floor and appearance of cleanliness.

**Table 1:** Participant Characteristics (*n* = 137) Collected during Survey 1

	%		Mean (SD)
Gender (Woman)	91%	Age	33.8 (9.2)
Literacy (Literate)	97%	Children	2.1 (1.2)
Secondary School or Higher	85%	Children (<6 years)	1.3 (0.5)
Participant or Spouse Works Near Mining	22%	Monthly expenses (USD)	211 (127)
Other Family member Works in Mining	38%	Monthly income (USD)	380 (216)
Respondent Employed Outside the Home	33%		

### 2.4 Home Soil Samples

With permission from participants, interviewers collected home dust or soil samples using a small dustpan and broom in the interior entryway of the house, or if this area was too clean, in another room (indoor home samples) during Surveys 1 and 2 conducted in July and September 2015, respectively. If it was not possible to collect sufficient material for analysis indoors, a soil sample was taken from just outside the front doorstep (outdoor home samples). Parents were asked the location of the sample from Survey 1 to determine the sample location during Survey 2. All home samples were sieved through a 1 mm sieve into 20‐ml scintillation vials and analyzed by XRF.

In Survey 1, home samples were collected from 145 households and 138 contained enough material (~1 g) for analysis by XRF (*n* = 54 indoor, *n* = 49 outdoor, and *n* = 35 unknown). During Survey 2, 139 home samples were collected, and 136 were analyzed (*n* = 50 indoor, *n* = 68 outdoor, and *n* = 18 unknown). Only 24 samples were confirmed to have been collected indoors both times.

### 2.5 Roadside Soil Mapping

Soil‐Pb concentrations were mapped in situ in July 2015 using the portable XRF analyzer in approximately 60‐m intervals along publicly accessible roads, pathways, parks, and railroad tracks. In 2016, we conducted additional XRF mapping where parents found a new hotspot (*n* = 37) and near homes not reached in 2015 (*n* = 23). The 2016 data expanded the coverage of the towns but also overrepresented the most contaminated areas.

### 2.6 Water Samples

At the request of the local community, 16 water samples were collected from drinking‐ and nondrinking water sources. Scintillation vials were rinsed three times in the source water before collecting a 20‐ml sample. Once in the lab, samples were acidified along with blank vials filled with MQ water. In August water samples were collected in duplicate and one was filtered through a 0.45‐μm nylon syringe filter. All ICP‐MS method and vial blanks contained <1 μg/L Pb and all vial blanks contained <0.1 μg/L.

### 2.7 Community Meeting

We presented information about soil‐Pb contamination and the health impacts of Pb at community meetings in each of the four towns. Central to these meetings was the presentation of a poster‐sized (2 m by 1 m) map of soil‐Pb levels in each town based on the roadside XRF mapping (Figure 1). Participants received a handout copy of the map (size A4) with key information discussed on the back. The map was posted in a central location in each town. In addition, in Corcona and Tornamesa, the towns with a hotspot of soil Pb >1,200 mg/kg, two more posters were placed near the main entry points to the contaminated areas.

The soil‐Pb maps were created in Google Earth, with colored pins indicating three health‐risk categories based on total‐Pb concentrations: low in green (<280 mg/kg Pb), medium in yellow (280 to 1,200 mg/kg Pb), and high in red (>1,200 mg/kg Pb; Figure 1). The 280 mg/kg Pb in soil threshold was set by doubling the Peruvian residential soil standard of 140 mg/kg (Ministerio del Ambiente (de Peru) (MINAM), 2013). For comparison, the U.S. EPA's soil hazard standard for bare soil in children's play areas is 400 mg/kg and for other residential areas is 1,200 mg/kg (U.S. EPA, 2001). The map also included the recommendations in Spanish to “protect your child–avoid contaminated areas” and advised, in high‐risk areas, “do not let children touch the contaminated soil,” and in medium risk areas “avoid that your children play on the ground,” and “avoid bringing this dirt to your home.”

The local municipalities provided the meeting space and informed residents about the meeting details. Such collaboration will be key for any future expansion of a soil‐Pb testing program. However, various delays in communication left little time between when the meeting was announced and when it took place. Even in the town where the mayor personally knocked on doors the morning of the meeting, only 4 of the 26 attendees were among the 19 participants in the study.

### 2.8 BLLs

After reviewing the soil‐Pb data in late 2015, the local health department (Dirección Regional Salud Ambiental) offered parents a blood‐Pb test for children 6 years old and younger. Health department workers were provided with a LeadCare II unit that they used to measure Pb in a small pin‐prick sample of blood with detection limit of 3.3 μg/dL (Meridian Bioscience, n.d.). The instrument is widely used for Pb monitoring across the United States because it can be used in nonspecialized doctor's offices that are not regulated for laboratory analysis. Children with a BLL > 9 μg/dL (*n* = 44) were offered confirmation testing, and 33 children returned for a second LeadCareII BLL measurement. We received BLL information for children whose parents signed additional BLL‐specific consent forms.

### 2.9 Field Procedure to Screen Soil Pb Used by Parents

In late April 2016, 11 parents from Corcona and nine parents from Tornamesa were invited to use a new field procedure to collect soil samples where their children play and screen them for Pb using a visual indicator (Figure S2). The 20 consenting parents were selected based on the blood‐Pb information of their children, provided by the health department, to capture soils where both the most exposed and least exposed children play. Parents attended a training session at the local health center to learn how to use the field procedure and borrowed a smartphone to record sample information.

Parents were asked to collect up to six soil samples: three from locations where their child currently played and three from alternative locations where their child could play. These alternative locations were included so that parents would already know of a safe alternative play area in case their current play area was determined to be contaminated. Parents collected the soil samples independently by sieving the soil through a basic kitchen sieve (~1‐mm mesh) and funnel into a 20‐ml scintillation vial. Using the SurveyCTO app (SurveyCTO, 2019), parents recorded geographic coordinates, a site photo, sample ID, and a brief description, occasionally with help from a family member or CREEH field staff. This information was stored by the app on the smartphone, without cellular connection, until it was uploaded to a central database by connecting to a wireless internet or cellular hotspot.

After collecting the soil samples, parents met with a researcher to analyze the samples using an earlier version of the field procedure for hazardous Pb described by Landes et al. (2019). This method is based on the 0.4 M glycine extraction (pH = 1.5) of Drexler and Brattin (Drexler & Brattin, 2007) and the U.S. EPA's standard bioavailability method for Pb (U.S. EPA, 2017). Parents were asked to add a small spoonful of soil (approximately 1 g) to 10 ml of the extraction solution in a scintillation vial and shake it for 30 s at least 45 min before meeting with a researcher. At the meeting parents shook the vial again for 30 s and allowed the soil to settle for 15 min, for a total extraction time of at least 1 hr. An aliquot of the extract was then decanted into the lid of the scintillation vial, and the tip of a white test strip impregnated with sodium rhodizonate (Plumbtesmo® MACHEREY‐NAGEL, #90602) was dipped into cap, allowing the liquid to rise via capillary action to 1 cm from the top.

The presence of Pb in the extract solution was determined by the color of the test strip and, in some cases, also the direct addition of sodium rhodizonate. If the test strip turned purple, the extract contained a high concentration of Pb. Comparison with ICP‐MS measurements had indicated that a purple test strip corresponded to >100 mg/L Pb in solution, or an equivalent of 1,000 mg/kg extractable Pb in soil given the dilution ratio of 1‐g soil in 10‐ml solution. If the test strip turned light purple or speckled purple, the extract was classified as containing a medium concentration of Pb. In this case, or if no color was seen, a toothpick tip of sodium rhodizonate powder was stirred into the cap with the decanted extract solution. The liquid visibly turned purple at concentrations >40‐mg/L Pb in solution, equivalent to 400 mg/kg extractable Pb in soil, thus confirming the presence of a midrange concentration of Pb in soil. After the color analysis with the parents, the liquid extract was filtered through a 0.2‐μm syringe filter into a clean 20‐ml scintillation vial by a researcher and immediately analyzed by XRF in the benchtop stand. After the fieldwork was completed, the extract Pb concentration was confirmed by ICP‐MS.

The main difference of the field procedure used in this study relative to the later version described in Landes et al. (2019) is that a test strip impregnated with sodium rhodizonate was inserted directly into the unfiltered solution after the extraction time of 1 hr instead of adding a gelatin capsule of sodium rhodizonate to the filtered extract (Landes et al., 2019). The procedure was later modified because we noticed that the Plumbtesmo® rhodizonate strips degraded with temperature and sunlight. By the end of the week, a test strip that had been in the field would no longer turn purple as it had at the beginning of the week. Since the sodium rhodizonate added to the extraction solution with a toothpick tip continued working throughout the week, it appears that the Plumbtesmo® strips are more susceptible to degradation.

### 2.10 Statistical Analysis

Statistical analyses were performed with R Studio 1.0.136 (RStudio Team, 2016) using R 3.5.1 (R Core Team, 2018). Since blood‐Pb levels and soil‐Pb concentrations were not normally distributed, Spearman's rank correlation was used. To check for significance between blood lead categories, we employed the package Tableone (Yoshida & Bohn, 2018), a chi‐square test for categorical variables, and a Kruskal‐Wallis test for nonparametric continuous data.

## 3 Results

### 3.1 Town Soil Samples

**Figure 3:**
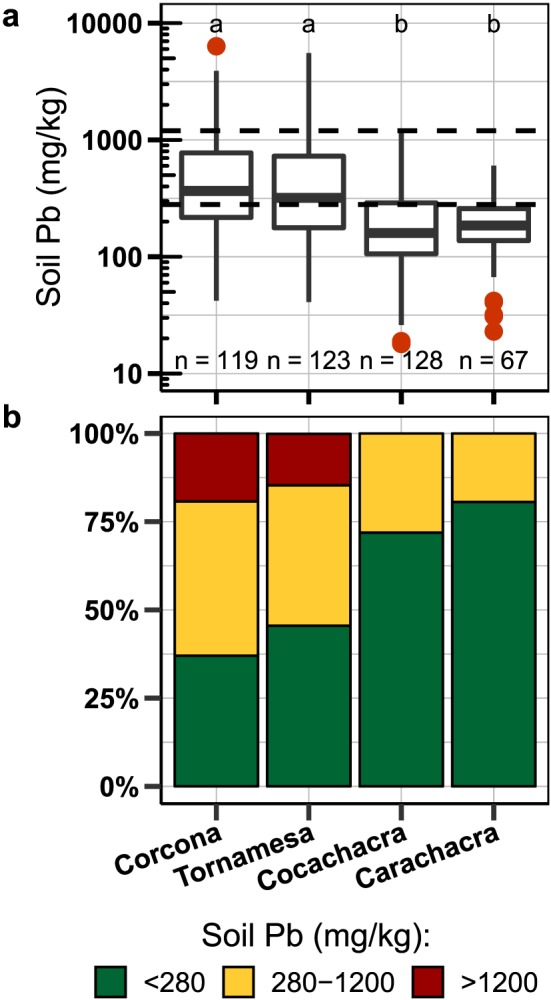
(a) Distribution of soil‐Pb concentrations per town from the 2015 roadside XRF mapping. Box created from the lower hinge (25%), median, and upper hinge (75%). Whiskers extend to 1.5* the interquantile range. (b) Proportion of samples per town below 280 mg/kg Pb, and above 1,200 mg/kg Pb. Different letters in (a) represent groups that are statistically different (*p*<0.05).

Concentrations of Pb measured at 437 locations during the roadside mapping in July 2015 range from 20 to 6,400 mg/kg Pb (mean ± 1 standard deviation (SD): 500 ± 750 mg/kg). The Pb levels in 44% of samples are above 280 mg/kg Pb, in 30% are above the U.S. EPA 400‐mg/kg hazard standard for bare soil where children play, and in 10% are above the U.S. EPA 1,200‐mg/kg hazard standard for other soil (Figure 3). The mapping identified two hotspots of soil Pb above 1,200 mg/kg: one in Corcona and one in Tornamesa (Figure 1). In Corcona, the hotspot stretches 1 km along a river levee and around an old mine visible in satellite images. In Tornamesa, the hotspot follows the railroad tracks for 0.2 km around a turntable. As a result, mean soil‐Pb is higher in the two towns with an identified hotspot, Corcona and Tornamesa (760 ± 90 and 680 ± 80 mg/kg, ±1 standard error of the mean (SEM)), than in the other two towns, Cocachacra and Carachacra (240 ± 20 and 200 ± 10 mg/kg, ±1 SEM; Figures 1 and 3, p<0.01).

By adding soil‐Pb information from 2016, including the samples parents collected and the additional in situ portable XRF mapping conducted to confirm high soil‐Pb, town‐level data is available for 586 soil samples. Of these samples, 51% are above 280 mg/kg, 37% are above 400 mg/kg, and 13% are above 1,200 mg/kg Pb. With the additional data for Corcona, where parents detected an additional hotspot, the mean Pb rises to 920 ± 80 mg/kg (±1 SEM) while remaining essentially unchanged in the other towns: Tornamesa (660 ± 60 mg/kg), Cocachacra (240 ± 20 mg/kg), and Carachacra (200 ± 10 mg/kg).

### 3.2 BLL

**Figure 4:**
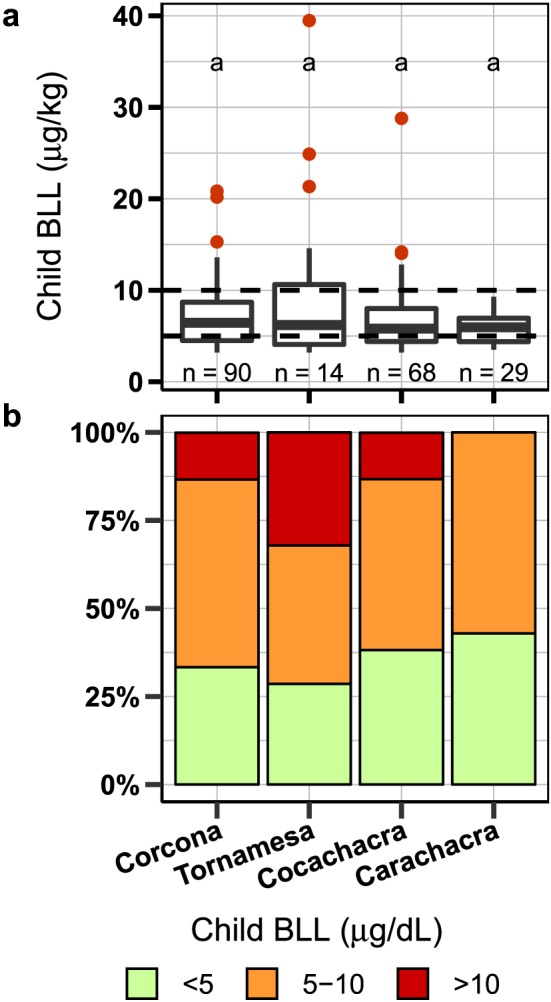
(a) Distribution of child BLL. Box created from the lower hinge (25%), median, and upper hinge (75%). Whiskers extend to 1.5 * the interquantile range. (b) Proportion of children with blood lead levels (BLL) over the reference levels of 5 and 10 μg/dL. BLL = blood lead levels. Similar letters in (a) represent groups that are not statistically different (*p*<0.05).

The arithmetic mean BLL measured in children across the four towns was 7.2 ± 4.5 μg/dL (mean ± SD) with a geometric mean of 6.4 ± 1.6 μg/dL (±1 geometric SD) (*n* = 200; Figure 4). Of these, BLL in 30 children (15%) were above 10 μg/dL, the Peruvian standard, and 130 (65%) were above 5 μg/dL, the CDC reference level. Of the 33 children tested twice, BLLs of 26 were confirmed above 9.0 μg/dL. The proportion of children with BLL >5 μg/dL varied from 57% to 71% across the towns. However, the proportion of children with BLL >10 μg/dL varied more noticeably between the towns with 13% in Corcona (*n* = 90), 32% in Tornamesa (*n* = 28), 13% in Cocachacra (*n* = 68), and 0% in Carachacra (*n* = 14). The arithmetic and geometric mean in these towns were, respectively, in Corcona, 7.1 ± 3.4 μg/dL (±1 SD) and 6.4 ± 1.5 μg/dL (±1 geometric SD), in Tornamesa, 9.2 ± 8.0 and 7.2 ± 1.9 μg/dL, in Cocachacra 6.8 ± 3.9 and 6.1 ± 1.6 μg/dL, and in Carachacra 5.9 ± 1.8 and 5.6 ± 1.4 μg/dL, and did not differ significantly (*p* = 0.593).

### 3.3 Water Samples

All water samples contain Pb concentrations below the Peruvian drinking water standard of 10 μg/L Pb and below the U.S. EPA Maximum Contaminant Level of 15 μg/L. All samples collected from drinking water taps, municipal springs or water sources contain less than 1 μg/L Pb. The highest Pb concentrations are found in nondrinking water sources, in concrete drainages along the road both uphill from Cocachacra (5 μg/L Pb unfiltered) and in Tornamesa (1.6 μg/L Pb unfiltered); water samples collected from the Riomac River, accessed in Tornamesa, contain 1.0 μg/L Pb in the unfiltered sample. Total acid‐dissolvable Pb concentrations in samples from a small creek running through Tornamesa are 3.0 μg/L (unfiltered) near the train tracks and, 2 months later, 1.5 μg/L (unfiltered) and 0.4 μg/L (filtered).

### 3.4 Parent Soil‐Pb Screening

Parents conducting soil screening with the field procedure identified six high‐Pb samples, five of which contain >500‐mg/kg extractable Pb measured in the field by XRF and correcting for dilution. Parents ranked 15 additional samples as medium, 13 of which contain >250‐mg/kg extractable Pb. Average extractable‐Pb concentration measured by ICP‐MS for samples ranked high is 610 ± 490 (±1 SD) mg/kg (*n* = 6), for samples ranked medium is 410 ± 280 mg/kg (*n* = 15), and for samples ranked low is 110 ± 80 mg/kg (*n* = 70). Average total soil‐Pb concentration, measured by XRF, for samples ranked high is 1,800 ± 1,240 mg/kg (±1 SD), for samples ranked medium is 870 ± 790 mg/kg, and for samples ranked low is 390 ± 340 mg/kg.

**Figure 5:**
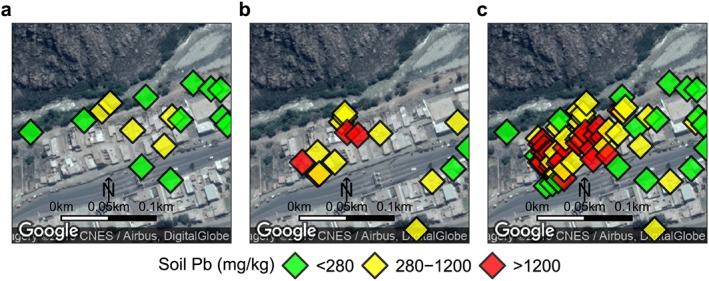
Progression from left to right of soil map for the area where parents identified a new hotspot by using a screening method for soil Pb. Pb concentrations are indicated by color in green (<280 mg/kg), yellow (280–1,200 mg/kg), and red (>1,200 mg/kg) for (a) roadside XRF mapping in 2015, (b) parent‐collected soil samples in 2016, and (c) confirmation in situ XRF mapping with data from (a) and (b).

The sampling and screening of soil conducted by parents revealed a new Pb hotspot. Three different parents collected high‐Pb soil samples from the same neighborhood in 2016 in an area in western Corcona that our 2015 XRF mapping did not identify as a hotspot. We confirmed and delineated this new Pb hotspot with additional XRF measurements (mean soil Pb: 1,800 ± 1,500 mg/kg, *n* = 37, (±1 SD); Figure 5). In addition, some parents collected samples near their work places, which included car wash areas. In one case, follow‐up in situ XRF mapping identified Pb concentrations up to 3,000 mg/kg, with average levels 900 mg/kg Pb (±180 SEM).

Across all samples collected by parents in areas where their children play, mean total soil‐Pb concentration is 560 ± 570 mg/kg (*n* = 59, ±1 SD), measured by XRF (median: 380, range: 20–3,500 mg/kg). Similarly, mean total soil‐Pb concentration in alternative play areas is 580 ± 760 mg/kg (*n* = 32, ±1 SD, median: 360, range: 60–4,200 mg/kg). For 11 parents, the mean total soil‐Pb concentration collected in alternative areas is lower than 20% of the mean of samples collected in current play areas; for 6 it is higher than 20%. While median Pb‐concentrations are below the U.S. EPA 400 mg/kg standard for both sets of samples, concentrations range widely.

#### 3.4.1 Proportion of Extractable Pb

**Figure 6:**
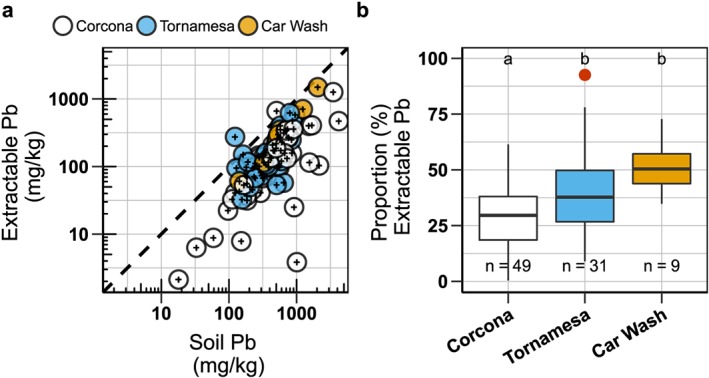
(a) Extractable Pb compared to total soil Pb from samples parents collected in areas where their children play and could play by town. Concentrations were measured by XRF for total Pb in soil and by ICP‐MS for extractable Pb. Error bars are 10%. (b) Proportion of extractable Pb is higher in Tornamesa and car wash areas than in Corcona. Two outliers that extracted more than 100%, after accounting for the dilution of 1‐g soil in 10‐ml extraction solution are outside the boundaries of plot b. Letters in (b) represent groups that are statistically different (*p*<0.05).

On average the field procedure extracted 38% ± 29% (±1 SD, *n* = 89) of the total soil Pb, after accounting for a soil‐to‐solution ratio of 1:10, and this proportion can be used to estimate the amount of Pb that would be released in the gastrointestinal system (Landes et al., 2019). Less Pb was extracted from samples in Corcona, 31% ± 20% (*n* = 49), than in Tornamesa, 45% ± 38% (*n* = 31), and in the car wash locations, 52% ± 11% (*n* = 9; *p* < 0.05) (Figure 6). A subset of 10 samples from these towns was also analyzed previously (Landes et al., 2019) by the standard bioaccessibility (IVBA) method from Drexler and Brattin (Drexler & Brattin, 2007) which extracted 60% ± 31% of the total soil Pb (*n* = 10). Additional correlation analysis of a suite of elements analyzed by XRF of the soil samples (Figure S8) further show geochemical differences between towns that could explain these differences. Comparison of Pb in different particle sizes, fine (<250 μm), large (>250 μm), and total (Figure S9), however, does not indicate a clear difference in the ratio of Pb in the fine fraction between towns. Concentrations of Pb in the smaller grain size (<250 μm) are not significantly higher than Pb concentrations in total sample. Pb concentrations in the larger grain size (>250 μm) particles contain are 45–90% the Pb concentrations in the total sample (Figure S9, Table S2).

#### 3.4.2 Parent Feedback

Parent feedback on using the field procedure revealed that parents found the procedure fairly easy to use (mean: 7.3 ± 1.9, ±1 SD, on a scale from 1 for very difficult to 9 for very easy). Parents were also fairly confident (mean: 3.6 ± 0.9 on a scale from 1 for not at all confident to 5 for very confident) that they could correctly gauge the color of the field‐procedure test strip and that they could collect the soil sample and add it to the extract solution (mean: 3.2 ± 0.9 on 1–5 scale). Parents reported some confidence in being able to do the entire procedure independently (mean: 3.0 ± 1.1 on 1–5 scale). When asked who they thought should collect the soil samples, parents selected, in decreasing order, themselves (*n* = 9), CREEH researchers (*n* = 4), the government (*n* = 4), a specialist for soil samples (*n* = 3), and the health department (*n* = 1). However, most parents stated that they preferred the field‐procedure color analysis be conducted by CREEH researchers (*n* = 9), followed by themselves (*n* = 6), a soil‐sampling specialist (*n* = 4), and the government (*n* = 2).

### 3.5 Home Soil Samples

For home samples confirmed to have been collected indoors, the mean Pb concentration is 210 ± 130 mg/kg (*n* = 54, range: 40–780 mg/kg Pb) during Survey 1 and 280 ± 210 mg/kg (*n* = 50, range: 30–1,500 mg/kg Pb) during Survey 2 (mean ± 1 SD). For those samples confirmed to have been collected outside of the home, mean Pb is 270 ± 200 mg/kg (*n* = 49, range: 40–1,080 mg/kg Pb) during Survey 1 and 280 ± 170 mg/kg (*n* = 68, 50–900 mg/kg Pb) during Survey 2.

**Figure 7:**
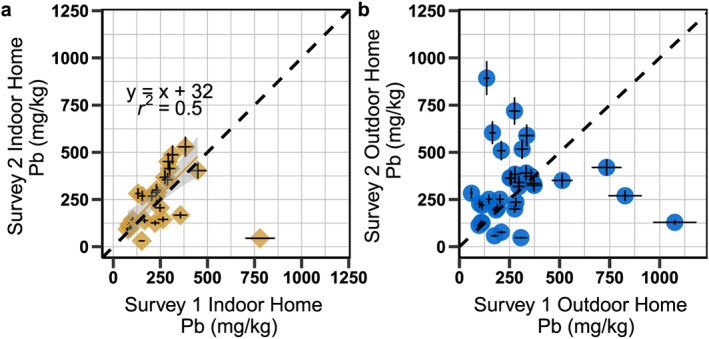
Lead concentrations in (a) indoor and (b) outdoor home samples measured by XRF in dust or soil collected during Survey 1 (July) and the Survey 2 (September). Many people swept and mopped daily and did not have enough dust inside the home for an indoor sample. Error bars are 10%. Best‐fit line calculated without the outlier.

Across all home samples, samples collected during Survey 1 correlate with samples collected during Survey 2 (Spearman's rank rho = 0.34, *p* < 0.001). However, when looking at only the 24 samples collected indoors during both surveys, the correlation is higher (Spearman's rho = 0.58, *p* = 0.004), which markedly improves when removing one outlier (Spearman's rho = 0.77 *p* < 0.001; Figure 7a). Concentrations of Pb for soils collected outdoors during both surveys do not correlate significantly (Spearman's rho = 0.31, *p* = 0.079; Figure 7b).

#### 3.5.1 Home Soil Samples in Relation to the Broader Environment

Average Pb concentrations (mg/kg Pb ± 1 SEM) in outdoor home soil samples are, in towns with a hotspot, Corcona and Tornamesa, (310 ± 40, *n* = 29 and 350 ± 10, *n* = 2), and in towns without a hotspot, Cocachacra and Carachacra, (210 ± 30, *n* = 17 and 130, *n* = 1). In indoor home samples, average Pb concentrations in Corcona and Tornamesa (200 ± 20, *n* = 23 and 190 ± 30, *n* = 10) are similar to Cocachacra and Carachacra (230 ± 40, *n* = 18, *n* = 17 and 290 ± 100, *n* = 3).

To assess the impact of local soil‐Pb variation on home soil‐Pb concentrations, we compared mean neighborhood soil‐Pb, calculated from the 5 to 9 measurements taken within 100 m of the homes in the 2015 roadside XRF mapping, to indoor and outdoor home samples from Survey 1 (Figure S3). There is a significant correlation of the 47 confirmed outdoor home samples paired with neighborhood soil data (Spearman's rho = 0.43, *p* = 0.003, *n* = 47). However, this is not the case for indoor samples paired with neighborhood soil data (rho = −0.04, *p* = 0.786, *n* = 49). By town, average Pb concentrations (mg/kg Pb ± 1 SEM) within 100 m of a home are higher in the towns with a hotspot, Corcona and Tornamesa (430 ± 40, *n* = 64 and 370 ± 30, *n* = 18), than in towns without a hotspot, Cocachacra and Carachacra (180 ± 10, *n* = 45 and 190 ± 10, *n* = 13, *p*<0.05). Including the 2016, soil‐Pb data increases the average in Corcona and Tornamesa (590 ± 60, *n* = 65 and 400 ± 40, *n* = 20) but does not change the correlation between 100 m soil‐Pb and home soil‐Pb samples.

### 3.6 Associations Between Blood and Soil‐Pb

**Figure 8:**
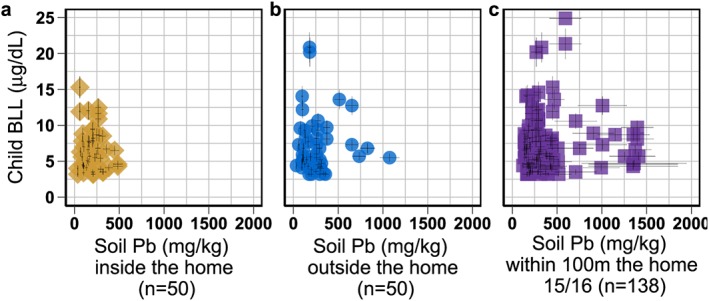
Child BLL with Pb levels from (a) soil or dust collected inside the home, (b) soil collected just outside the home, and (c) the average of soil samples within 100 m of the home measured by roadside XRF mapping. All child BLL shown that if a parent had more than one child, then the parent's soil samples are repeated. Pb concentrations measured by XRF. Error bars are 10% for soil Pb inside and outside of the home. For soil Pb within 100 m of the home, error bars are the standard error of all the soil measurements averaged. BLL = blood lead level.

There is no correlation between an individual child's BLL and Pb concentrations in home soil samples, whether confirmed indoor samples (Spearman's rho: 0.02, *p* = 0.887, *n* = 50) or outdoor samples (Spearman's rho: −0.04, *p* = 0.786, *n* = 50; Figure 8). There is also no correlation between BLL and mean Pb of the 2015 soil samples collected within 100 m of the home (Spearman's rho: −0.03, *p* = 0.734, *n* = 135). This does not change when including soil‐Pb data from the 2016 follow‐up in the mean Pb concentrations within 100 m (Spearman's rho: 0.06, *p* = 0.520, *n* = 138). Soil‐Pb concentrations are not significantly associated with categorical BLL of the youngest child (Table 2).

**Table 2:** Soil Pb and Survey 1 Parent Responses and Observations by Blood‐Pb Category of the Youngest Child Per Parent (*n* = 110)

Soil Pb factors	BLL >10		BLL 5‐10		BLL <5		ANOVA/Kruskal–Wallis test		
	Mean (n)	Median (IQR 25%–75%)	Mean (n)	Median (IQR 25%–75%)	Mean (n)	Median (IQR 25%–75%)	*p* value		
Indoor House Pb (mg/kg)	215 (4)	210 (131–293)	207 (22)	203 (145–259)	209 (14)	181 (102–309)	*p* = 0.999	nonnorm	kruskal.test
Outdoor House Pb	317 (6)	229 (182–454)	301 (18)	200 (119–296)	231 (12)	265 (153–309)	*p* = 0.886	nonnorm	kruskal.test
Soil Pb 100 m	385 (16)	283 (239–456)	417 (51)	297 (173–431)	419 (39)	285 (191–433)	*p* = 0.889	nonnorm	kruskal.test
Soil Pb 200 m	420 (17)	341(197–448)	500 (51)	361 (282–727)	416 (3)	337 (197–420)	*p* = 0.41	nonnorm	kruskal.test
Soil Pb 100 m–2015 Only	345 (14)	273 (220–333)	287 (51)	286 (173–345)	394 (39)	263 (191–368)	*p* = 0.73	nonnorm	kruskal.test
Soil Pb 200 m–2015 Only	382 (15)	286 (197–380)	392 (51)	315 (259–377)	402 (39)	286 (197–410)	*p* = 0.785	nonnorm	kruskal.test
Fieldkit Soil Pb in Areas Where Children Play	413 (6)	377 (300–505)	728 (7)	528 (406–780)	560 (6)	518 (318–726)	*p* = 0.524	nonnorm	kruskal.test
Fieldkit Extractable Pb in Areas Where Children Play	185 (6)	140 (125–224)	253 (7)	216 (116–284)	140 (6)	129 (89–198)	*p* = 0.524	nonnorm	kruskal.test
Fieldkit Total Soil Pb All Samples	625 (6)	558 (386–829)	684 (7)	523 (423–744)	474 (6)	433 (296–619)	*p* = 0.572	nonnorm	kruskal.test
Fieldkit Extractable Pb in All Areas	237 (6)	155 (123–282)	234 (7)	216 (126–265)	126 (6)	131 (90–173)	*p* = 0.324	nonnorm	kruskal.test
	BLL >10		BLL 5–10		BLL <5				
Observational	n (%)		n (%)		n (%)		*p* value		
How Dusty Was Home							*p* = 0.854	chi‐square test	
Not At All	2 (15)		12 (22)		10 (29)				
A Little	7 (54)		30 (56)		18 (51)				
Very	4 (31)		12 (22)		7 (20)				
Shoes Dirty							*p* = 0.035	chi‐square test	
Not At All	4 (25)		28 (53)		25 (68)				
A Little	8 (50)		18 (34)		11 (30)				
Very	4 (25)		7 (13)		1 (3)				
Clothing Dirty							*p* = 0.005	chi‐square test	
Not At All	5 (31)		35 (65)		27 (73)				
A Little	7 (44)		16 (30)		10 (27)				
Very	4 (25)		3 (6)		0 (0)				
How Many Children <6 Playing on the Ground							*p* = 0.053	chi‐square test	
None	7 (44)		24 (44)		23 (62)				
25%	5 (31)		26 (48)		13 (35)				
50%	3 (19)		4 (7)		1 (3)				
75%	1 (6)		0 (0)		0 (0)				
Participant Observed Child							*p* = 0.368	chi‐square test	
Not At All	3 (19)		18 (33)		15 (42)				
A Little	4 (25)		16 (30)		11 (31)				
Very	9 (56)		20 (37)		10 (28)				
Observed Shoes Removed							*p* = 0.801	chi‐square test	
None	16		53		36				
25%	0 (0%)		1 (2%)		1 (3%)				
Observed Clothes Changed							*p* = 0.368	chi‐square test	
None	16		52		37				
25%	0 (0%)		2 (4%)		0 (0%)				
Other Factors	Mean (n)	Median (IQR 25%–75%)	Mean (n)	Median (IQR 25%–75%)	Mean (n)	Median (IQR 25%–75%)			
Spending Time in Likely Contaminated Areas	2.1 (14)	2.1 (1.0–3.0)	1.8 (51)	1.6 (1.0–2.5)	1.7 (36)	1.4 (1.0–2.2)	*p* = 0.45	nonnorm	kruskal.test
Avoiding Likely Contaminated Areas	1.5 (14)	1.0 (0.5–2.5)	1.9 (51)	2.0 (1.0–2.5)	2.0 (36)	2.0 (1.0–3.0)	*p* = 0.38	nonnorm	kruskal.test
Picking Up the Child in Likely Contaminated Areas	2.6 (14)	2.3 (1.3–4.0)	2.3 (51)	2.0 (1.0–3.3)	2.2 (36)	1.8 (1.0–3.5)	*p* = 0584	nonnorm	kruskal.test
Child Sucking Thumb	2.8 (16)	2.5 (1.9–4.0)	3.0 (54)	3.0 (2.0–4.0)	3.0 (37)	3.0 (2.5–4.0)	*p* = 0.799	nonnorm	kruskal.test
Child Wash Hands Before Eating and After Playing Outside	3.1 (16)	3.7 (2.6–4.0)	3.2 (54)	3.3 (2.7–4.0)	3.3 (37)	3.7 (2.7–4.0)	*p* = 0.896	nonnorm	kruskal.test
Removing Shoes When Coming Home (1 = Almost Never, 5 = Almost Always)	1.3 (16)	1.0 (1.0–1.0)	1.7 (54)	1.0 (1.0–2.0)	1.5 (37)	1.0 (1.0–2.0)	*p* = 0.2	nonnorm	kruskal.test
Change Clothing When Getting Home From Work	2.5 (16)	2.5 (1.0–3.1)	2.9 (54)	3.0 (2.0–4.0)	2.9 (37)	3.0 (1.0–4.0)	*p* = 0.512	nonnorm	kruskal.test
	*n* (%)		*n* (%)		*n* (%)				
Saw a Poster of Soil Pb	10 (71)		35 (65)		26 (70)		*p* = 0.816	chi‐square test	
Live in Town With Hotspot	10 (63)		32 (59)		59.5% (37)		*p* = 0.972	chi‐square test	
Do You Paint Your Home With “Artesanal” Paint?	2 (13)		7 (13)		1 (3)		*p* = 0.229	chi‐square test	
Do You Buy Informal Gasoline?	3 (33)		2 (22)		0 (0)		*p* = 0.240	chi‐square test	
Do You Have Metal Pipes?	0 (0)		1 (2)		0 (0)		*p* = 0.434	chi‐square test	
Do You Use Traditional Medicines?	12 (75)		31 (57)		24 (65)		*p* = 0.416	chi‐square test	
Demographic Factors	*n* (%)		*n* (%)		*n* (%)				
Gender (Woman)	15 (94)		47 (87)		35 (95)		*p* = 0.429	chi‐square test	
Literacy (Read and Write)	15 (94)		52 (96)		36 (97)		*p* = 0.177	chi‐square test	
Secondary School or Higher	12 (75)		44 (82)		33 (89)		*p* = 0.472	chi‐square test	
Participant or Spouse Works in Mining	3 (19)		10 (19)		7 (19)		*p* = 0.750	chi‐square test	
Other Family Member Works in Mining	6 (38)		16 (30)		14 (38)		*p* = 0.674	chi‐square test	
Family Member Recycles Batteries	0 (0)		4 (7)		3 (8)		*p* = 0.513	chi‐square test	
Family Member Recycles Electronic Waste	0 (0)		4 (7)		1 (3)		*p* = 0.365	chi‐square test	
	Mean (n)	Median (IQR 25%–75%)	Mean (n)	Median (IQR 25%–75%)	Mean (n)	Median (IQR 25%–75%)			
# Rooms in Household	2.6 (16)	2 (1.8–4)	2.9 (54)	2 (2–3)	3.5 (37)	3 (2–5)	*p* = 0.139	nonnorm	kruskal.test
How Many m2 Is House	95.6 (16)	85 (75–120)	94.5 (54)	90 (52.5–120)	102 (37)	90 (60–100)	*p* = 0.914	nonnorm	kruskal.test
Age of Parent	32 (16)	30 (27–38)	34 (54)	32 (27–39)	32 (37)	31 (24–38)	*p* = 0.525	nonnorm	kruskal.test
Monthly Expenses (PEN)^a^	612 (16)	550 (388–675)	628 (54)	600 (363–738)	576 (37)	600 (300–800)	*p* = 0.892	nonnorm	kruskal.test
Monthly Income (PEN)^a^	1069 (16)	1100 (775–1350)	1182 (54)	1000 (800–1500)	1142 (37)	1000 (750–1500)	*p* = 0.913	nonnorm	kruskal.test
Age of Youngest Child	2.44 (16)	2.0 (2.0–3.0)	3.0 (54)	3.0 (2.0–4.0)	2.7 (37)	2.6 (0.9–4.0)	*p* = 0.272	nonnorm	kruskal.test

a 1 Peruvian Nuevo Soles (PEN) = 0.322 USD.

Similarly, there is no correlation between child BLL and Pb in soil samples collected by the parent in areas where the child currently played (Spearman's rho: −0.01, *p* = 0.953, *n* = 29) (Figure S4a). If examined by category of BLL, the distribution of Pb concentrations in the soil samples collected by parents do not differ noticeably between the families with child BLL categorized as low (<5 μg/dL), medium (5–10 μg/dL) or high (<10 μg/dl; Table 2 and Figure S5a). The differences are larger when comparing the extractable Pb to these BLL categories, especially the absence of a highly extractable Pb in the lowest BLL category of <5 μg/dL (Figure S5b). However, BLL is not correlated with the extractable Pb measured by ICP‐MS either (Spearman's rho: 0.13, *p* = 0.512, *n* = 29; Figure S4b).

### 3.7 Associations Between Blood and Interview Information

**Figure 9:**
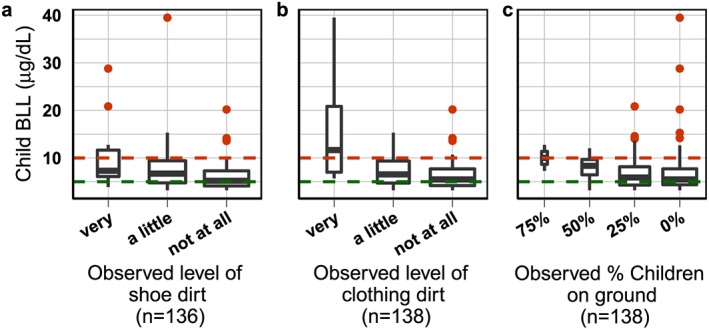
Child BLL by (a) observed level of shoe dirt, (b) observed level of clothing dirt, and (c) observed percent of children playing on the ground. All child BLL shown that if a parent had more than one child, then the parent's soil samples are repeated.

Blood‐Pb levels for all children show significant associations with two observations recorded by the interviewer during Survey 1, before any child BLL information had been obtained (Figure 9 and Table 2). Interviewers recorded whether the parents' clothing and shoes appeared dirty on a scale from 1 (not at all) to 4 (very much); the highest recorded value was 3 (very). Both observations of the cleanliness of parents' shoes (Spearman's rho: 0.29, *p* = <0.001, *n* = 138) and clothing (Spearman's rho: 0.31, *p* = <0.001, *n* = 136) are significantly correlated and associated with categorical child BLL groups (*p* = 0.035 and *p* = 0.005; Table 2). Finally, the proportion of children observed playing on the ground neared significance across categorical child BLL groups (*p* = 0.053) and correlation (Spearman's rho: 0.16, *p* = 0.067, *n* = 138).

Child age, which is often associated with BLL, is not significantly correlated (Spearman's rho: −0.03, *p* = 0.756, *n* = 143) when looking at each child individually (Figure S6). However, parent education level is correlated with BLL when looking at each child individually, thus repeating parent education levels for parents with more than one participating child (Spearman's rho: −0.19, *p* = 0.029, *n* = 138; Figure S7). This association is just not significant when looking at only the youngest child of each parent (Spearman's rho: −0.15, *p* = 0.107, *n* = 110) and not at all across BLL category (*p* = 0.472; Table 2). Neither of these interpretations change when removing the outlier of the child BLL near 40 μg/dL. Other factors such as self‐reported behaviors avoiding contaminated areas, washing hands, or removing shoes when arriving at home were not associated with child BLL (Table 2). Child BLL was also not associated with type of water pipes used, purchasing informal gasoline, using artisanal paints, using traditional medicines, a parent working in mining, extended family working in mining, a family member recycling batteries or electronic waste, income, or size of home (Table 2). In some cases, answers were practically uniform across all participants; for instance, only one home reported having metal pipes.

For these analyses, results from Survey 1 are used because meeting attendance was low across all towns; only 14 of the 68 community meeting attendees were parents in the study. In total, only 10% of study participants attended the community meeting. However, 70% reported seeing at least one poster with the map of Pb contamination and 44% reported seeing the map at least once a day (Table 2). Parents ranked the map as easy to understand, on a scale from 1 (very difficult) to 5 (very easy), with an average of 4.1 ± 0.9. When asked why they did not attend the meeting, 59% said they did not know about the meeting, and another 7% said they did not have enough information about the exact time and location of the meeting. Other common reasons included being unavailable due to work (10%), family obligations (7%), and other activities (12%).

## 4 Discussion

### 4.1 Causes and Variations of Pb Contamination

Pb concentrations in soil in these four mining‐impacted towns vary greatly, and importantly, high levels of contamination are concentrated in hotpots, confirmed by XRF as soil >1,200 mg/kg Pb. The Corcona hotspot extended from the gates of a mining school situated on a former mine that produced barite, galena, and sphalerite and along the levee protecting Corcona from the nearby River Rimac. The levee was reportedly constructed in part with waste material from the nearby mine. The Tornamesa hotspot was centered around the railroad turntable. However, we did not find the expected hotspots along the rest of railroad running through the four towns, even though uncovered flatbed railroad cars historically passed through carrying ore concentrate from the La Oroya smelter and mining areas. Replacement of railroad ties, which we observed in Cocachacra, likely covered past contamination along other sections. The railroad section in Tornamesa may remain contaminated because train cars spend more time above these track sections as they are uncoupled and recoupled around the turntable.

Parents collecting soil samples in 2016 from areas where their children play and screening the samples using a field procedure identified a new hotspot of Pb contamination that we had missed in our 2015 mapping. While we had stayed on publicly accessible paths and roads, these new highly contaminated samples came from and around the excavated sites of new home construction. The excavation likely brought up underlying mine tailings that had been capped with low‐Pb surface soil. It is unclear whether we missed this hotspot in 2015 by staying on public‐access ways or because there was less construction. These areas were not fenced off and we observed children playing in them.

Since the hotspots may represent different types of Pb contamination, it is not surprising to see variations in extractable Pb (Figure 6), which can be used to calculate bioaccessible Pb, or the proportion absorbed by the gastrointestinal system. Bioaccessibility of Pb depends on its chemical form, size fraction, and the soil matrix (Casteel et al., 2006; Ruby et al., 1999). Lead from mining sources is often less bioaccessible than Pb from leaded‐gasoline, smelters, or urban areas (Appleton et al., 2013; Casteel et al., 2006; Landes et al., 2019; Ruby et al., 1999). The lead contamination in Corcona is likely from the nearby mine and probably less extractable for that reason. Lead found in Tornamesa along the railroad turntable is from the ore concentrate carried by the trains and thus more processed and more extractable. Correlation (Figure S8) further confirm these geochemical differences between the soil samples between towns that could be relative to the different forms of contamination. The difference in extractability is not due to differences in Pb in the fine particle size, as no clear difference is seen between Corcona and Tornamesa (Figure S9). Reassuringly, the Pb concentrations in the total soil sample (<1 mm) appear to be representative of particles more likely to stick to hands and clothes: Concentrations of Pb in the smaller grain size (<250 μm) are only slightly higher than Pb concentrations in the total sample.

### 4.2 Child BLLs in Relation to Previous Observations

Geometric mean BLLs in children under 6 years of age in the four communities (6.4 μg/dL) were markedly higher than BLLs in U.S. children (0.84 μg/dL in 2013–2014; Tsoi et al., 2016). In 65% of 200 children, BLLs were above the CDC reference value of 5 μg/dL, while only 0.5% of U.S. children were over this value in 2013–2014 (Tsoi et al., 2016). On average BLLs in 15% of children were over 10 μg/dL, although Tornamesa stands out with almost twice as high a proportion (32%, *n* = 28). These proportions, however, are lower than most of the previously reported Peru BLLs above 10 μg/dL, including 58% of 335 children in La Oroya (Red Salud, 2014) and 53% of 356 children in Cerro de Pasco in 2008 (Conklin et al., 2008). Subsequent studies in Cerro de Pasco show a decrease from 2012, when BLLs for 44% of 2,671 children exceeded 10 μg/dL (Dirección Regional Salud Ambiental Pasco, 2012), to 2014 when only 17% of 757 children exceeded this level (Ministerio de Salud, Perú (MINSA), 2014). Compared to our study towns, both Cerro de Pasco and La Oroya are larger industrial cities with a history of Pb contamination and exposure from open‐pit mining and ore smelting, respectively.

Geometric mean (GM) BLL in Corcona (6.4 ± 1.5 μg/dL), Tornamesa (7.2 ± 1.9 μg/dL), Cocachacra (6.1 ± 1.6 μg/dL), and Carachacra (5.6 ± 1.4 μg/dL) were comparable to those reported in Cartagena, Colombia (4.7 μg/dL, *n* = 189 school children age 6.9 ± 0.1) in 2004 (Olivero‐Verbel et al., 2007), as well as the 6.8 μg/dL found in Mexico in 1,207 adolescents (ages 1–14) and the 4.9 μg/dL found in 2,001 infants (<1 year of age) since 2000 (Caravanos et al., 2014). At the other end of the spectrum, BLLs in these Peruvian towns were far below the ones measured in the Mitrovica region in the United Nations Administered Province of Kosovo between 1999 and 2007, where geometric mean BLL of 98 children was 50.3 μg/dL in 1999–2004 and dropped to 25.6 μg/dL for 16 children tested in 2006–2007 (Brown et al., 2010).

### 4.3 Environmental Factors Impacting BLL

Somewhat to our surprise, we do not see a correlation between BLL and soil‐Pb or dust‐Pb concentrations, whether within 100 m of the home (*p* = 0.734), in outdoor home samples (*p* = 0.786), or in indoor home samples (*p* = 0.887). Similarly, we do not see a correlation between BLL and mean soil Pb in samples collected by parents where their children played. Our sample size is limited however, and while association between soil Pb and BLL is difficult to determine (Laidlaw & Filippelli, 2008), it has been reported elsewhere (Conklin et al., 2008; Mielke & Reagan, 1998; von Lindern et al., 2003; Zahran et al., 2013). In addition, Lanphear et al. (1998) found home dust‐Pbloadings to be the major contributor to child BLL (Lanphear et al., 1998) and had previously found that dust‐Pb loading were much more associated with child BLL than dust‐Pb concentrations alone (Lanphear et al., 1995). Dust‐Pb loading data, for example, Pb per area, was not collected in this study, and the indoor home soil samples sieved to <1 mm and may not reflect exposure to Pb from dust as well as wipe samples, vacuum samples, or samples sieved to <250 or <150 μm, the fraction that sticks to children's hands. Homes that were too clean and did not have a sufficient amount of dust to be collected for analysis by XRF do not have indoor home data, thus biasing the data set.

The association between BLL and total soil‐Pb concentrations could be obscured by variations in the bioaccessibility of Pb, which are different between the towns and their contamination sources. Based on this, it was expected that extractable‐Pb concentrations in samples collected by parents where their children played, might better reflect child BLL. However, while there were no samples with highly extractable Pb in the lowest BLL category below 5 μg/dL (Figure S5), extractable Pb did not correlate significantly (rho: 0.13, *p* = 0.512, *n* = 29) with BLL (Figure S4b and Table 2), potentially due to the small sample size. Comparing aggregate town BLL to soil Pb supports the difference in bioaccessible Pb or other factors varying impacting child BLL at a town level. For example, in Tornamesa the mean soil Pb is 680 mg/kg and BLLs in 32% of children >10 μg/dL (GM BLL 7.2 μg/dL), whereas in Corcona the mean soil Pb is 760 mg/kg and BLLs in only 13% of children >10 μg/dL (GM BLL of 6.4 μg/dl; Figures 3 and 4).

Past studies have found that dust lead concentrations and loading rates were difficult to accurately measure and reflected the myriad of socioeconomic and behavioral factors (Laidlaw & Filippelli, 2008; von Lindern et al., 2003). Samples collected at a single point in time may not accurately reflect the average conditions a child is exposed to because of spatial and temporal variability of Pb in soil. This is indicated, for instance, by the lack of correlation of soil Pb outside the home between Surveys 1 and 2 (Figure 7b). And while soil Pb within 100 m is correlated (rho = 0.43, *p* = 0.003, *n* = 47) with outdoor home Pb, the relationship is far from consistent (Figure S3). This suggests that soil‐Pb is so variable that sampling does not properly represent soil‐Pb concentrations that children are exposed to. Furthermore, since data on how much time a child spends where is not available, the soil we tested around a child's home may not represent the Pb levels to which a child is most exposed. When parents collected soil samples where their children played, at least two (of 20) traveled more than 1km to collect samples, in one case from a grandparent's home in the neighboring town. Some children attend school in different towns while young children may spend most of the day with their parent at work: in one case soil‐Pb concentrations near a car wash area were up to 3,000 mg/kg. Cars and trucks coming down from the mountain potentially include mining‐related vehicles covered with Pb‐contaminated dust.

Children are potentially exposed to Pb from a variety of sources, and soil Pb or home dust‐Pb may not be the primary drivers of child BLL in this region. Drinking water is, however, likely not a contributing factor as water analysis from the public drinking water sources and supply all samples contain <1 μg/L Pb, and all but one home had plastic pipes. We did observe a local parent collecting water from the stream in Tornamesa that contained up to 3 μg/L Pb (unfiltered), who clarified that while she knew this was not drinking water, she would consider it safe to drink after boiling. In these communities, child BLL was also not associated with parent survey responses about the use of informal gasoline, artisanal paints, or traditional medicines or a parent or family member working in mining, battery recycling, or electronic waste (Table 2).

Because the proportion of elevated child BLL varies by both soil and dust Pb levels, we ran multiple scenarios of the Integrated Exposure Uptake Biokinetic Model (IEUBK) for Lead in Children, varying input soil‐Pb concentrations but keeping all other default values consistent. This model was used to determine the U.S. EPA hazard level of 400 and 1,200 mg/kg and estimates that at soil‐Pb concentrations less than or equal to 500 mg/kg, the probability that a single child's BLL would be greater than 10 μg/dL is 1 to 5% depending on dust‐Pb concentrations (U.S. EPA, 2001). The IEUBK model outputs estimates that BLLs in 0.4% of children ages one to six exceed 10 μg/dL when soil Pb is 200 mg/kg, and 28% exceed this level when soil Pb is 760 mg/kg. While this model is not inconsistent with our findings for Tornamesa, (mean soil Pb: 680 mg/kg, 32% >10 μg/dL), it overestimates the number of children who would exceed 10 μg/dL in the other towns of Corcona (soil Pb: 760 mg/kg, 13% >10 μg/dL), Cocachacra (soil Pb: 240 mg/kg, 13% >10 μg/dL), and Carachacra (soil Pb: 200 mg/kg, 0% >10 μg/dL).

Local conditions could be different from the basic assumptions of the IEUBK model, including that children spend more time outside in these Peruvian towns compared to the United States, that some homes had dirt floors, and that low soil moisture conditions could increase soil resuspension. We therefore tested the sensitivity of the IEUBK model to varying the soil ingestion rate to see if this could account for the difference in predicted and measured child BLLs. Increasing the soil and dust intake rate 50% from the default raises the GM BLL from 2.9 to 3.8 μg/dL for children exposed to soil Pb of 200 mg/kg and from 7.6 to 10.4 μg/dL for children exposed to soil at 760 mg/kg Pb. These ingestion rate settings applied with town mean soil‐Pb and home soil‐Pb concentrations result in the following BLLs: Carachacra (predicted 5.1 vs. measured 5.6 μg/dL), Tornamesa (7.4 vs. 7.2 μg/dL), Corcona (8.0 vs. 7.2 μg/dL), and Cocachacra (4.8 vs. 6.8 μg/dL). These variations indicate that there could be differing factors impacting BLL even between our study towns, such as the bioaccessibility of Pb in soil.

### 4.4 Parent and Behavioral Factors Impacting BLL

Socioeconomic and behavioral factors are well known to impact child BLLs (Jones et al., 2009; Mahaffey et al., 1982; Pirkle et al., 1994). Individual behaviors can impact the child BLL by interrupting the exposure pathway, and indeed, in this study, child BLLs were most significantly associated and correlated with the cleanliness of the parents' shoes (*p* < 0.001) and their clothing (*p* < 0.001). This indicates the importance of parents' behavior in the transfer of soil and dust Pb to children. While these observations could reflect interviewer bias, neither the participant nor the interviewer had knowledge of child BLL at this time. These factors are robust enough that they remain significant when considering only the youngest child per parent across BLL category (Table 2). While parent cleanliness is associated with child BLL, self‐reported changing of clothing and shoes after coming home from work is not nor is the appearance of the dustiness of the home (Table 2). It appears that the interviewer observations, rather than self‐reported actions, better capture behaviors that are associated with child BLL, and perhaps represent more standardized or less biased indicators of behavior. The 2008 CDC report from Cerro de Pasco showed that increased BLLs were significantly associated with demographic factors such as child age and having a family member who worked in mining (Conklin et al., 2008). In our study, the only demographic factor that significantly correlates with child BLL is parent education level (*p* =0.029) when looking at all children, repeating parent education levels in the case of multiple children from the same parent. Parent income, number of rooms in a home, and child age are not significantly associated. However, even parent education level is not significantly correlated when looking at only the youngest child of each parent (*p* = 0.107) or across BLL category (*p* = 0.472; Table 2). This could reflect the small sample size of *n* = 110 when taking the youngest child per parent and *n* = 138 when taking each child individually and repeating parental factors. Finally, child age and individual variations in child nutritional status, especially iron‐deficiency, can also impact BLL (Kordas, 2017); nutritional information is not available for this study.

### 4.5 Limitations and Challenges

The outcome of this study is consistent with the limitations highlighted by the review by Laidlaw and Filippelli (2008) for determining the association between soil Pb and blood Pb, including misclassification of exposure due to limited point samples and low sample size. While our study started out with a reasonable sample size, the number of paired child BLL data with Pb concentrations from indoor soil, outdoor soil, and play areas is very limited, especially across BLL category. In addition, not all factors that can impact child exposure to Pb, including spatial and temporal variations in total and extractable Pb in soil, indoor home soil and/or dust samples and size fraction, time children spend in which locations, child hand‐to‐mouth behavior, and child iron‐deficiency, were adequately captured in this study.

### 4.6 Implications for Parents Collecting Environmental Data to Reduce Child Pb Exposure

A larger, more systematic intervention should be conducted to quantify the impact on child exposure of combining parent health education with parent‐led environmental sampling and testing, given that (a) parent cleanliness was related to child BLL, (b) parents successfully collected soil samples in this pilot study, (c) parents preferred they be the ones to collect soil samples, and (d) the high variation in total and extractable Pb concentrations in soil. There is considerable scope for testing the effectiveness of a more participatory approach, because educational interventions have been shown to reduce child BLL but are not always effective (Hilts et al., 1998). Covering, or replacing, high‐Pb soil with clean soil has also been shown to reduce child BLLs (Laidlaw et al., 2017; Lanphear et al., 2003; von Lindern et al., 2003). Reducing indoor dust‐Pb loading, has also been connected to decreases in child BLL, especially at higher BLLs (Braun et al., 2018; Haynes et al., 2002), although it appears there are diminishing returns at lower BLL (Braun et al., 2018; Haynes et al., 2002).

For scaling up this approach, parents could collect soil samples where their children might be exposed and return these samples to a local health center or school, where trained staff analyzes the samples with or for parents. In the case that such an intervention seeks to create town‐wide maps of soil‐Pb, one challenge could be recording the geolocation of the samples with a touchscreen on a smartphone. In our study, younger parents or those accustomed to touchscreen phones had no difficulties, and we anticipate that this challenge would decrease with time as smartphones and touchscreens become more common in towns throughout the world.

## 5 Conclusions

The four small mining‐impacted towns studied here contained hotspots of high concentrations of Pb in soil and children with elevated BLLs. Hundreds of similar towns exist in Peru and around the world where children may be exposed but no environmental or exposure data are collected. This study shows that parents are motivated and capable of collecting soil samples using a simple field procedure to help identify these potential areas of hazardous soil Pb. Parents found an additional hotspot of Pb greater than 1,200 mg/kg that had not been identified by our original soil mapping. While there was no clear relationship between these parent‐collected soil samples, or other soil samples, and child BLL, parent shoe and clothing cleanliness was related to BLL and appears to link parents' behavior to the transfer of soil and dust Pb to children. Parent cleanliness may therefore be a valuable target of educational interventions, possibly as part of soil sampling and testing to identify and reduce exposure to hotspots of Pb contamination.

## CONFLICT OF INTEREST

The authors declare no conflicts of interest relevant to this study.

## Supporting information

Supporting Information S1
