# Supplementary material for: Does Involving Parents in Soil Sampling Identify Causes of Child Exposure to Lead? A Case Study of Community Engagement in Mining‐Impacted Towns in Peru
Source: Geohealth. 2019 Aug 23;3(8):218–36. doi: 10.1029/2019GH000200 (PMC7007120; doi:10.1029/2019GH000200)
Supplement: Supplementary file 1 — Supporting Information S1 [file GH2-3-218-s001.docx]

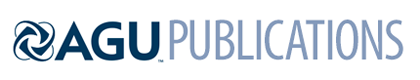


*GeoHealth*

Supporting Information for

**Does involving parents in soil sampling identify causes of child exposure to lead? A case-study of community engagement in mining-impacted towns in Peru.**

Franziska C. Landes^1,2*^, Jennifer Inauen^3^, Johny Ponce-Canchihuamán ^4,5^, Kathie Markowski^1^, Tyler K. Ellis^1^, Alexander van Geen^1^

^1^Lamont-Doherty Earth Observatory, Columbia University, Palisades, New York 10964, USA, ^2^Department of Earth and Environmental Sciences, Columbia University, New York, New York 10027, USA, ^3^Institute of Psychology, University of Bern, 3012 Bern, Switzerland, ^4^Center for Research in Environmental Health, Lima, Lima-11, Peru, ^5^Universidad Peruana Cayetano Heredia, Facultad de Salud Publica y Administracion, Lima, Lima-31, Peru

Corresponding author: Franziska Landes (fcl2115@columbia.edu)

**Contents of this file**

Figures S1 to S9.

Table S1 to S2.

Figure S1. Blank-corrected XRF measurements of Pb in extract solution collected in the field immediately after extraction compared to ICP-MS measurements of the same solution in the laboratory several months later. The dotted line is the one-to-one line.


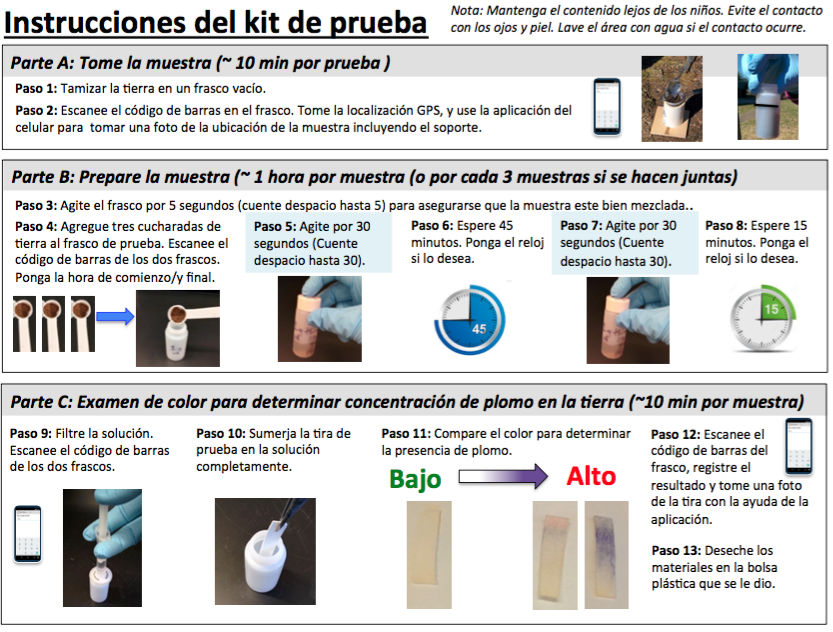


Figure S2. Instruction sheet including all field procedure steps for parents collecting and analyzing soil samples. Parents received this or a similar version that focused only on the steps Part A and Part B that they were completing on their own.

Figure S3. Pb concentrations within 100m of the home compared with home soil samples collected (a) indoors and (b) outdoors. Closed symbols indicate samples collected during Survey 1, and open symbols indicate samples from Survey 2. Lead concentrations in dust and soil measured by XRF in July-September 2015. Error bars of home Pb samples are 10% and error bars of the soil Pb within 100m of the home is the standard error of the number of measurements.

Figure S4. Child BLL levels with (a) mean soil Pb from samples parents collected where their children play, and (b) mean extractable Pb from those same samples. All children BLL are shown: if a parent had more than one child, then parents' soil samples are repeated. Small black triangles inside the circles indicate that a child had a sibling. Pb concentrations measured by XRF for total Pb in soil and by ICP-MS for extractable Pb. Error bars shown are 10% for both.


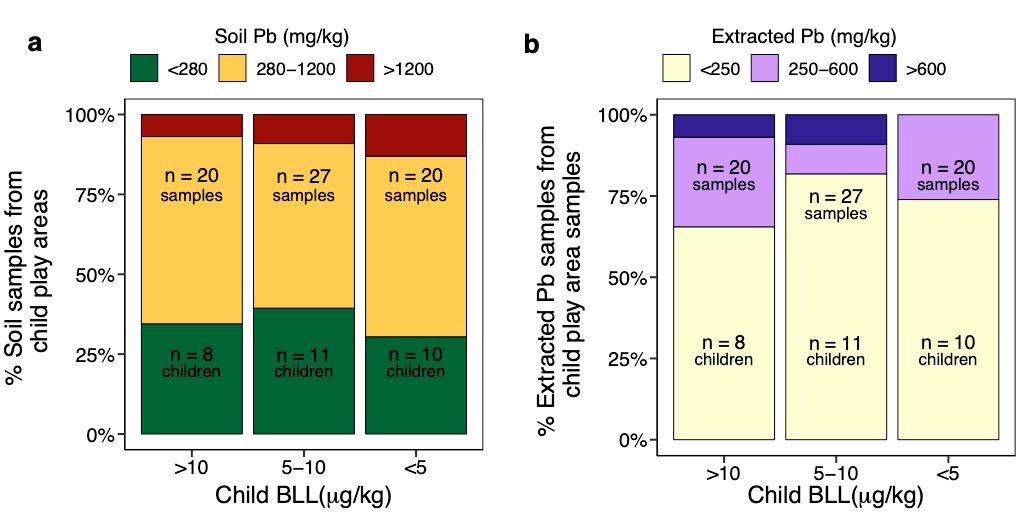
Figure S5. Parent field kit samples by child BLL category for (a) total Pb in soil samples and (b) extractable Pb from the same soil samples. All child BLLs are shown: if a parent had more than one child, then the parents' soil samples are repeated. Pb concentrations measured by XRF for total Pb in soil and by ICP-MS for extractable Pb.

Figure S6. Child BLL by age. All children with BLL information shown: if a parent had more than one child, then both children are shown.

Figure S7. Child BLL by parent education including (a) all children with BLL information, in which case if a parent had more than one child, then both children are shown and parent education level is repeated; and (b) only the BLL information from the youngest child of each parent.

**

Figure S8. Matrix of spearman rank correlations of elements analyzed by the XRF in soil samples. Accurate XRF analysis of elements was confirmed by comparing to XRF analysis of NIST soil standards. Gray X indicates the correlation was not significant. Values below the limit of detection (LOD) were included as 1/2 their LOD value. Arsenic (As) was also significantly correlated, but excluded due to a known interference between the As the Kα emission energy of 10.543 and the Pb Lα emission energy of 10.551, if one element is more than 10x the other (https://www.olympus-ims.com/en/applications/xrf-technology-analysis-arsenic-lead-soil/). Variations between towns are visible, reflecting different geochemical make-ups. In particular, Pb is significantly correlated with zinc and copper. In samples from the homes built on mine tailings (tailings) and in samples from Corcona, Pb also correlates with strontium and barium, which is consistent with the presence of the barite mine in Corcona. The most notable difference between Corcona and Tornamesa is the opposite direction of the correlation between Pb and calcium and strontium, as well as the much stronger association with barium.

Figure S9. Pb concentrations in the total soil sample, in the fine particle size (<250 μm), and large particle size (<250 μm) (Table S2). Samples are ordered in decreasing ratio of Pb (mg/kg) in the fine particle size to Pb in the total size fraction. Numbers on top of error bar indicate the ratio of Pb in the fine or large particle size to Pb in the total size fraction. Pb measured by XRF, and error bars are the standard deviation of three repeat measurements, shaking end-over-end between analyses in an attempt to capture heterogeneity. No clear difference seen in the ratio of Pb in the fine fraction between Corcona and Tornamesa or soil samples from the neighborhood built on mine tailings (tailings) in Corcona and the homes near the train tracks (train) in Tornamesa (p=0.926).

Table S1. Drinking water and non-drinking water sample results

| **Drinking water sources** | | |  |  |  |
| --- | --- | --- | --- | --- | --- |
|  | **sample_id** | **Town** | **Collected** | **Lead (μg/L)** | **Filtered/unfiltered** |
|  | DW_01 | Tornamesa | July 2015 | 0.05 | unfiltered |
|  | DW_02 | Tornamesa | August 2015 | 0.59 | unfiltered |
|  | DW_03 | Tornamesa | August 2015 | 0.19 | filtered |
|  | DW_04 | Tornamesa | August 2015 | <0.1 | unfiltered |
|  | DW_05 | Tornamesa | August 2015 | <0.1 | filtered |
|  | DW_06 | Tornamesa | August 2015 | 0.41 | unfiltered |
|  | DW_07 | Tornamesa | August 2015 | <0.1 | filtered |
|  | DW_08 | Corcona | July 2015 | 0.17 | unfiltered |
|  | DW_09 | Corcona | August 2015 | <0.1 | unfiltered |
|  | DW_10 | Corcona | August 2015 | <0.1 | filtered |
|  | DW_11 | Corcona | August 2015 | 0.05 | unfiltered |
|  | DW_12 | Corcona | August 2015 | <0.1 | filtered |
|  | DW_13 | Cocachacra | July 2015 | 0.61 | unfiltered |
|  | DW_14 | Cocachacra | August 2015 | 0.34 | unfiltered |
|  | DW_15 | Cocachacra | August 2015 | 0.15 | filtered |
|  | DW_16 | San Bartolome | July 2015 | 0.17 | unfiltered |
|  | DW_17 | Carachacra | July 2015 | 0.62 | unfiltered |
|  |  |  |  |  |  |
| **Non-drinking water sources** | | |  |  |  |
|  | NDW_01 | Tornamesa | July 2015 | 1.00 | unfiltered |
|  | NDW_02 | Tornamesa | July 2015 | 1.64 | unfiltered |
|  | NDW_03 | Tornamesa | July 2015 | 3.03 | unfiltered |
|  | NDW_04 | Tornamesa | August 2015 | 1.45 | unfiltered |
|  | NDW_05 | Tornamesa | August 2015 | 0.39 | filtered |
|  | NDW_06 | Cocachacra | July 2015 | 5.09 | unfiltered |

Table S2. Pb concentrations in different grain sizes.

| **Sample Type** | **Pb (mg/kg) in fine (<250 μm)** | **% total** | **Pb (mg/kg) in large (>250 μm)** | **% total** | **Pb (mg/kg) in total** | **Sample Description** |
| --- | --- | --- | --- | --- | --- | --- |
| Tornamesa | 335 | 104% | 180 | 56% | 322 | Tornamesa - other home |
| Corcona | 528 | 106% | 243 | 49% | 500 | Corcona - other home |
| Corcona | 522 | 106% | 305 | 62% | 491 | Corcona - other home |
| Mine | 4254 | 107% | 2725 | 69% | 3959 | Corcona - soil in front of mine |
| Tailings | 4348 | 109% | 2685 | 67% | 3992 | Corcona - home on mine tailings |
| Train | 371 | 109% | 197 | 58% | 339 | Tornamesa - home near train tracks |
| Tailings | 1267 | 115% | 515 | 47% | 1104 | Corcona - home on mine tailings |
| Train | 455 | 116% | 268 | 68% | 391 | Tornamesa - home near train tracks |
| Tailings | 1163 | 119% | 884 | 90% | 981 | Corcona - home on mine tailings |
| Train | 393 | 120% | 146 | 45% | 328 | Tornamesa - home near train tracks |
| Tornamesa | 320 | 121% | 175 | 66% | 266 | Tornamesa - other home |
| Tailings | 725 | 138% | 403 | 77% | 526 | Corcona - home on mine tailings |
